# Guanosine and Deoxyinosine Structural Analogs Extracted from Chick Early Amniotic Fluid Promote Cutaneous Wound Healing

**DOI:** 10.3390/ijms241612817

**Published:** 2023-08-15

**Authors:** Mashaal Ahmad, Jia Yu, Sha Cheng, Zara Ahmad Khan, Yan Luo, Heng Luo

**Affiliations:** 1 Department of Anatomy, College of Basic Medical Sciences, Guizhou Medical University, Guiyang 550031, China; mashaal-ahmad@gmc.edu.cn; 2 State Key Laboratory of Functions and Applications of Medicinal Plants, Guizhou Medical University, Guiyang 550031, China; yujia@gzcnp.cn (J.Y.); chengsha@gzcnp.cn (S.C.); 3 The Key Laboratory of Chemistry for Natural Products of Guizhou Province, Chinese Academy of Sciences, Guiyang 204236, China; 4 State Key Laboratory of Oncogenes and Related Genes, Institute for Personalized Medicine, School of Bio-Medical Engineering, Shanghai Jiao Tong University, Shanghai 200240, China; khanzaraahmad@sjtu.edu.cn; 5 The Center for Translational Medicine, Guizhou Medical University, Guiyang 550031, China

**Keywords:** amniotic fluids, wound healing, growth factors, natural compounds, skin regeneration

## Abstract

Wound healing is a complex, dynamic process supported by a myriad of cellular events that must be tightly coordinated to efficiently repair damaged tissue. These wounds are a significant socioeconomic burden due to their high prevalence and recurrence, which is why the phenomenon of wounds has also been labeled as a “Silent Epidemic”. Most of these wounds become “chronic”, with around 15% of them remaining unresolved 1-year post incidence, which results in a prolonged yet avoidable burden to patients, families, and the health system. In this experimental study, we tried to purify the potent components in chick early amniotic fluid (ceAF) and applied these components to the wound healing mechanism. We first subjected ceAF to a series of purifications, including an HPLC purification system along with ion-exchange chromatography technology to purify other potential components. Upon narrowing down, we found two structural analogs: guanosine and deoxyinosine. We performed these components’ cell scratch and trans-well migration assays to validate the accurate dosage. We also assessed these components via topical administration on the skin of murine model wounds. For this, we randomly divided C57BL/6 (all black, male, 5 weeks old) mice into groups. The wound model was established through excising the skin of mice and treated the wounds with different fractions of guanosine and deoxyinosine continuously for 8–10 day intervals. Once the healing was complete, the skin was excised to determine the inflammatory response and other biochemical parameters of the healed skin, including epidermal thickness, collagen density, macrophages, and neutrophil infiltration at the wounded site. Quantitative real-time PCR and immunoblot assays were performed to determine active gene expression and protein expression of proinflammatory molecules, growth factors, and cytokines. All these findings support our data indicating the promising healing properties of guanosine and deoxyinosine isolated from ceAF.

## 1. Introduction

The process of evolution has created our skin as a highly adaptive and multi-functional system which protects us from the daily exposure of ultraviolet, chemical, and physical challenges. However, these harsh environmental factors can still cause damage to the skin the form of injuries, chronic wounds, burns, and ulcers. Although, there is no surprise in the promising healing abilities of our skin which possess sophisticated reparative processes [1]. Even though the skin comprises this set of promising innate healing abilities, several cellular mechanisms, depending on the type of injury response, could become attenuated, thereby weakening and delaying the healing of wounds. This disruption of the skin barrier is often resulted due to pathogen invasion systematic changes in the pathology, including aging or uncontrolled diabetes [2]. Unfortunately, these chronic wounds, including pressure sores, venous ulcers, and foot ulcers, are a major area of concern, which is increasing exponentially on a global level [3]. Different phases of the wound healing process include inflammation, new tissue formation, and tissue remodeling [4,5]. The healing mechanism begins immediately post-injury via the release of several growth factors, cytokines, and low molecular weight components from the serum of injured blood vessels and from degrading platelets due to injury. The proliferative phase begins with the proliferation and migration of keratinocytes at the wound’s edges, followed by the proliferation of dermal fibroblasts in the surroundings. As a result, these cells eventually migrate to the provisional matrix and deposit an extensive extracellular matrix. The resulting wound connective tissue is also known as granular tissue due to having a granular appearance of several capillaries. In the end, a shift from granulation tissue to mature scar occurs, identified as continued collagen synthesis and catabolism of collagen. The scar tissue lacks appendages and is mechanically insufficient, having sebaceous glands, hair follicles, and sweat glands. The scarring process can be excessive and more drastic as hypertrophic scars and keloids [6].

For many years, certain therapeutic interventions, such as using natural compounds, herbs, and components extracted from natural sources, have made their mark in the field of regeneration medicine. For example, the use of amniotic fluids extracted from humans [7], chickens [8], mucus extracted from snails [7] and herbs extracted from different plants have been used extensively to cure different types of anomalies, including cancer treatment [9], diabetes, and wound healing. The research community realizes that 47 amino acids and related substances were identified in chicken embryos’ amniotic fluid, plasma, and allantoic fluid; mainly, there are other compounds [6,10]. These amino acids, lipids, and structural components are vital in developing embryos and organogenesis. Many studies have explored the capability of these molecules derived from the early amniotic fluid of chickens for tissue repair and regeneration for the treatment of cardiac [11], renal, and ischemic injuries [12], and hepatic and cutaneous wounds [11].

Amniotic fluid contains mostly water, comprising a solute content of essential nutrients and immune factors along with a series of other growth factors and stimulants, including lipids, peptides, amino acids, and nucleic acid building blocks [13]. These components, in principle, can be separated using cutting-edge technologies, such as high performance liquid chromatography (HPLC), mass spectrometry, separation based on molecular weight, hydrophilic or hydrophobic affinity chromatography, and other purification techniques. Recent studies have shown that purines are critical in controlling organogenesis and embryo development. In order to control the different factors of signal transduction that confirm proper embryo development, changes in the expression of enzymes controlling the metabolism of purines (adenosine deaminase and ectonucleotidase) and receptors encoding the message are among the crucial steps [14]. The P1, P2Y, and P2X receptor subtypes are involved in developing different organs, including blood vessels, the brain, the heart, and skeletal muscle [14]. In the maturation and growth of excitable cells, including neural cell genesis, the release of intracellular Ca^2+^ is regulated by mesoderm cells through the involvement of specific P2Y purinergic receptors, which are mainly governed through ATP [15].

This study explored the potential therapeutic benefits obtained by purifying chick early amniotic fluid (ceAF) through cutting-edge technologies, including HPLC-coupled reverse-phase chromatography and mass spectrometry. We found structural analogs of guanosine and deoxyinosine in ceAF and applied these components separately to cutaneous wound healing (both in vitro and in vivo). Our study depicts the promising therapeutic potentials of ceAF and validates our previous studies, where we used ceAF as a healing compound without any purifications [8,9].

## 2. Results

### 2.1. ceAF Plays a Vital Role in the Migration of Cells

To understand whether ceAF stimulates the proliferation and migration of cells, we first utilized ceAF as a whole and tested it on the keratinocyte cell line HaCaT. As indicated in our previous study [8], ceAF plays a crucial role in cell migration compared to the control group but in a dosage-dependent manner. We also performed in vivo mice modeling and supplemented ceAF on cutaneous wounds. ceAF exhibited promising healing of cutaneous wounds as different biological parameters validated via H&E staining, Masson’s trichrome staining, immune blotting, and quantitative real-time PCR. Our findings indicated the promising therapeutic abilities of ceAF against cutaneous wound healing.

### 2.2. Refinement of the Peaks Obtained from HPLC and Reverse-Phase Chromatography Analysis

**Figure 1:**
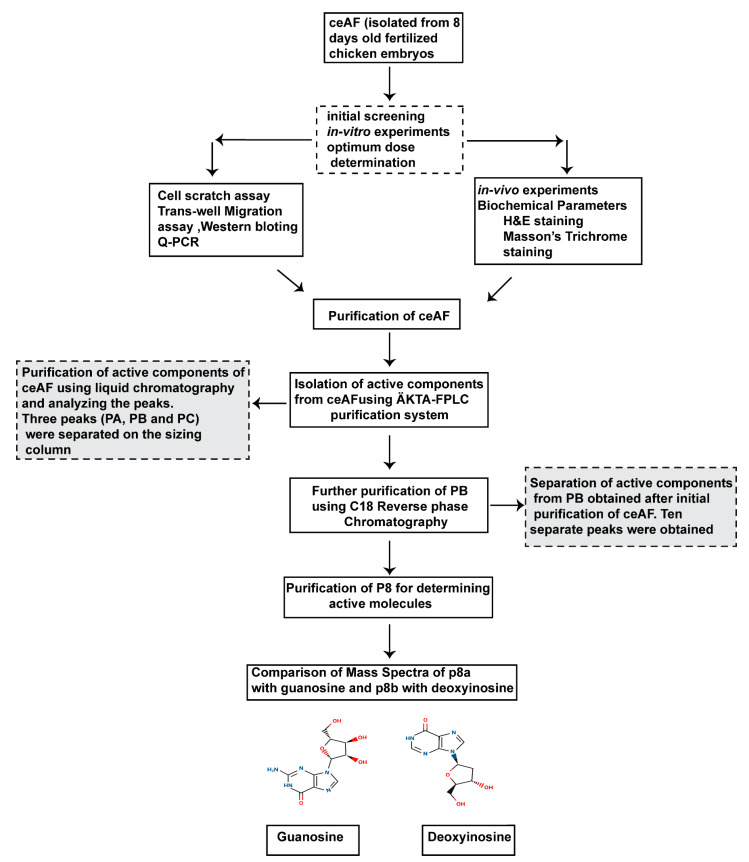
Schematic illustration of the extraction and purification of the active components, guanosine and deoxyinosine, from native ceAF. We first determined the biological activity of ceAF as a whole. Later, once it was established that ceAF possesses optimum wound healing properties, ceAF was subjected to several purification techniques, including the ÄKTA-FPLC purification system and reverse-phase chromatography, which led us to the structural analogs of guanosine and deoxyinosine when compared with the mass spectra of the chemical structures of these chemicals.

Once we purified the components of ceAF via HPLC and reverse-phase chromatography, 10 distinct peaks were obtained. P8 peaks showed optimum activity closer to native ceAF (Figure 1). Thus, we focused on further purifying P8 using an HPLC procedure coupled with mass-spectra analysis (QTof, Vion IMS, Waters) and obtained two sub-peaks; p8a and p8b (see Supplementary Figures S1 and S2). We also compared the mass spectra of potentially similar molecules; it was speculated that the two highly enriched possible active components (highest peaks) are structural analogs of guanosine (p8a) and deoxyinosine (p8b) (Figure 1). Guanosine (chemical formula: C_10_H_13_N_5_O_5_) and deoxyinosine (chemical formula: C_10_H_12_N_4_O_4_) (Figure 1) are products of highly active nucleotide metabolism and, in principle, can be derived from fast-growing embryos that may also serve signaling roles for fast cell growth. Indeed, compared with analytically pure guanosine and deoxyinosine, the two highest peaks within p8a and p8b can be deemed to be almost identical to these two molecules (Figure 1). This prompted us to directly assess the wound healing capabilities of these two molecules.

### 2.3. The Half-Maximum Effective Concentration of Guanosine and Deoxyinosine

**Figure 2:**
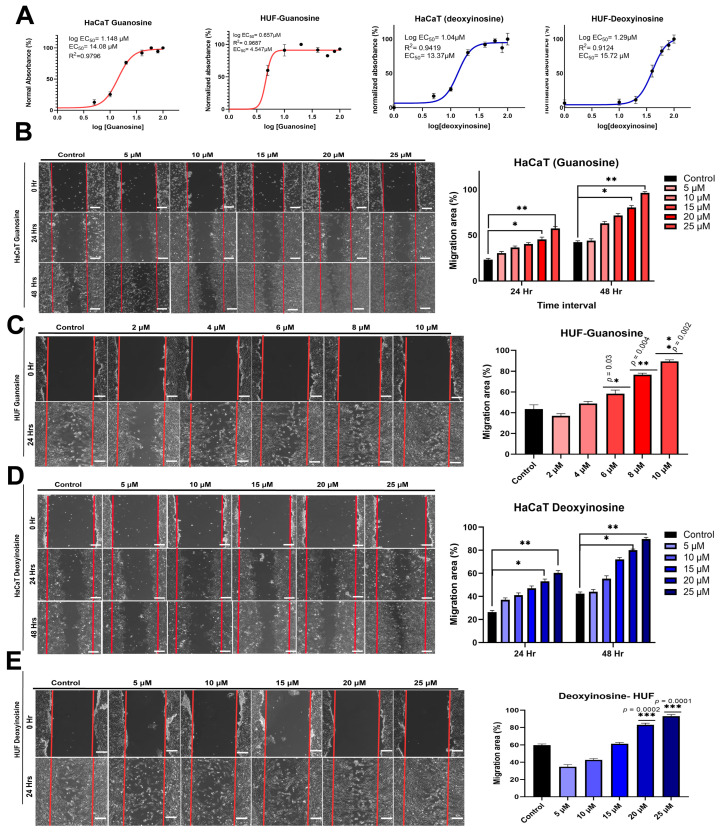
Guanosine and deoxyinosine CCK8 responsive curves on HaCaT and HUF cells. HaCaT and HUF cells were treated with guanosine and deoxyinosine (**A**). Guanosine promotes the migration and wound closure of HaCaT and HUF cells in a wound scratch assay. Representative images of HaCaT and quantification (**B**–**E**). The images were taken immediately post-scratch induction (0 h) and later after 24 h and 48 h, respectively, in the presence of different dosages of guanosine versus the control (10% FBS without treatment). Red lines indicate the initiatory areas of cells without migration. The in vitro wound healing assay showed that guanosine could be one component that signals cells to migrate when compared with the control group. The scalebar represents 100 μm. Data have been represented as mean ± SD, where the treated groups were found to be significantly different from the control group using the one-way ANOVA, whereas * *p* < 0.05, ** *p* < 0.01, *** *p* < 0.001.

Given that guanosine and deoxyinosine may be significant (and potent) components of ceAF that may mimic the wound healing capacity of ceAF, we first explored optimal doses, i.e., half-maximum effective concentrations (EC50), that were determined using two cell lines, including keratinocyte (HaCaT) and fibroblast (HUF) cell lines for in vitro studies (CCK8 assays). For the HaCaT cell line, the EC50 for guanosine was 14.08 μM; for deoxyinosine, it was 13.37 μM. Similarly, for HUF cells, the EC50 for guanosine was 4.54 μM; for deoxyinosine, it was 15.7 μM (Table 1; Figure 2A). This indicates the promising proliferative abilities of components extracted from ceAF with the minimum concentration required to achieve maximum output.

### 2.4. Guanosine and Deoxyinosine Support Wound Healing In Vitro

Once the effective concentrations of both components were determined, we established dosages encompassing the EC50 values of these chemicals. Since the keratinocytes treated with guanosine resulted in an EC50 value of 13.37 μM, we made a titration for HaCaT cells ranging from 5, 10, 15, 20, and 25 μM, respectively. A series of migration and proliferation experiments were performed. The scratch assay was performed to determine whether guanosine and deoxyinosine contribute to the fast migration of cells. Fibroblasts took 24 h to fill the gap (Figure 2C,E), whereas keratinocytes took 48 h to migrate to the scratched area (Figure 2B,D). Also, for both components, the optimum concentration at which the maximum number of cells migrated in the shortest interval was between 20 and 25 μM for the HaCaT and HUF cells. However, since the EC50 of guanosine for the HUF cell line was the lowest, the optimum dosage value ranged between 8 μM and 10 μM.

### 2.5. Guanosine and Deoxyinosine Extracted from ceAF Promote the Migration of Keratinocytes and Fibroblasts

**Figure 3:**
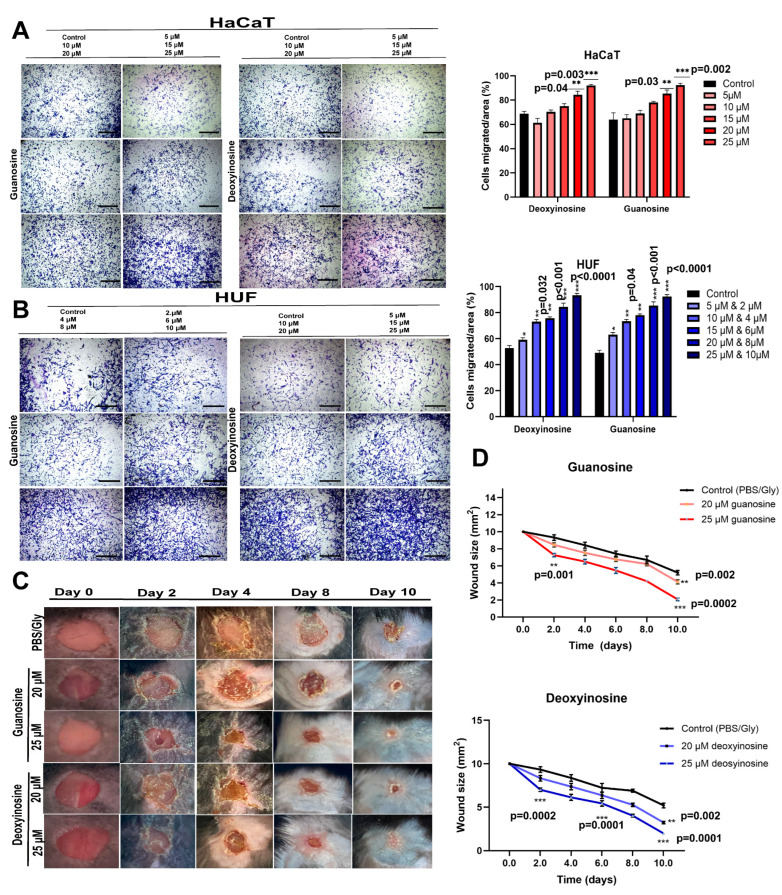
Trans-well migration assay for keratinocytes and fibroblasts treated with deoxyinosine and guanosine ((**A**,**B**); quantification on right panel, scalebar represents 100 μm). Different dosages of deoxyinosine and guanosine were supplemented with fibroblasts and keratinocytes. Later, the relative number of cells was evaluated using ImageJ software. All experiments were repeated at least three times. Significance was measured using a one-way ANOVA followed by a Dunnett’s multiple comparisons test to compare the control group (10% FBS) with other groups. * *p* < 0.05, ** *p* < 0.01, *** *p* < 0.001. Accelerated wound healing by means of treatment with guanosine and deoxyinosine isolated from ceAF. Wounds receiving no treatment (control), with different concentrations of guanosine and deoxyinosine (20 and 25 μM) at the time of wound induction (Day 0) till the complete healing (Day 10) are shown in (**C**). Fractions of wounds healed with 25 μM of guanosine and deoxyinosine at different time intervals are shown in (**D**). Statistical significance was evaluated with a two-way ANOVA with a Bonferroni post-hoc test. (*n* = 5/group) and ** *p* < 0.01, *** *p* < 0.001.

A trans-well cell migration assay was performed to investigate the effects of guanosine and deoxyinosine on keratinocytes and fibroblasts. Results were quantified using ImageJ software (version 1.53 q) to count the relative number of cells. As revealed in Figure 3A,B, along with quantification adjacent to the panels, the migration abilities of cells were significantly enhanced under the induction of guanosine and deoxyinosine at specified dosages. In contrast, the migration abilities of cells below the threshold dose were significantly decreased compared with the control. This indicates the promising migration of cells at a particular range obtained through the EC50 assay when treated with guanosine and deoxyinosine in a dosage-dependent manner.

### 2.6. Guanosine and Deoxyinosine Accelerate the Wound Healing Process in Mice

**Figure 4:**
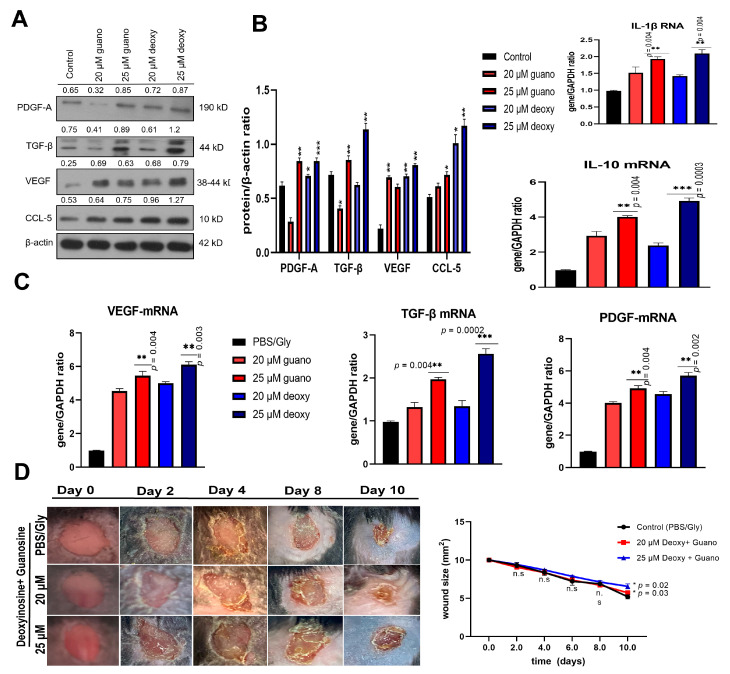
Guanosine and deoxyinosine promote the expression of growth factors and cytokines. (**A**) Immunoblots of growth factors, cytokines, and chemokines are shown, where β-actin has been used as an internal control (**B**). Quantitative real-time PCR of genes, including IL-1β, IL 10, PDGF, and VEGF mRNA, has been shown, where the housekeeping gene GAPDH has been used as an internal control. (**C**) Statistical analysis of the mRNA expression in wound tissue examined using qPCR, where the housekeeping gene GAPDH has been used as an internal control. The Student’s *t*-test was employed for this analysis where *n* = 5/group and *** *p* < 0.001, ** *p* < 0.01, and * *p* < 0.05. (**D**) Guanosine and deoxyinosine synergistically delay the healing of wounds. Wounds receiving no treatment (control) with different concentrations of guanosine and deoxyinosine (20 and 25 μM) at the time of wound induction (Day 0) till complete healing (Day 10) are shown. Wound size reduction in the time course is shown on the right side. Statistical significance was evaluated with a two-way ANOVA followed by a Bonferroni post-hoc test. (*n* = 5/group) and * *p* < 0.05.

A murine model for observing cutaneous punctured wound healing was established after topical administration of optimum concentrations of guanosine and deoxyinosine. Treating wounds with guanosine and deoxyinosine resulted in fastidious healing in adjacent skin two days after wound induction, indicating that the healing of wounds was a dose-dependent event. Daily administrations of 20 μM and 25 μM guanosine and deoxyinosine (in the form of a cocktail mixed with PBS and glycerol to attain a thick density) to wounds resulted in accelerated wound closure compared with untreated wounds (Figure 3C). No additional effect on wound healing was demonstrated through increasing the dose by more than 25 μM in both components. Also, for both analytically pure components, dosages below 15 μM did not offer efficient healing capabilities; mixing two chemicals did not exhibit cooperative efficacies, suggesting that they might trigger similar signaling pathways under study (Figure 4D).

### 2.7. Guanosine and Deoxyinosine Promote the Signaling of Growth Factors during Wound Healing

The inflammation-related growth factors, cytokines, and signaling of chemokines were analyzed in RNA (isolated from tissue samples after euthanizing the animals). The expression of anti-inflammatory IL-10 was increased in guanosine and deoxyinosine, where a 25 μM dose was used, as shown in Figure 4C. Following these growth factors promoting wound healing, PDGF and VEGF expression levels were also significantly increased. Overall, guanosine and deoxyinosine promoted the anti-inflammatory response along with the secretion of growth factors to promote fast wound healing, supporting the promising therapeutic role of ceAF (Figure 4A,B).

### 2.8. Guanosine and Deoxyinosine Promote Anti-Inflammatory Responses and Increased Disposition of Collagen Fibers in Wound Healing

**Figure 5:**
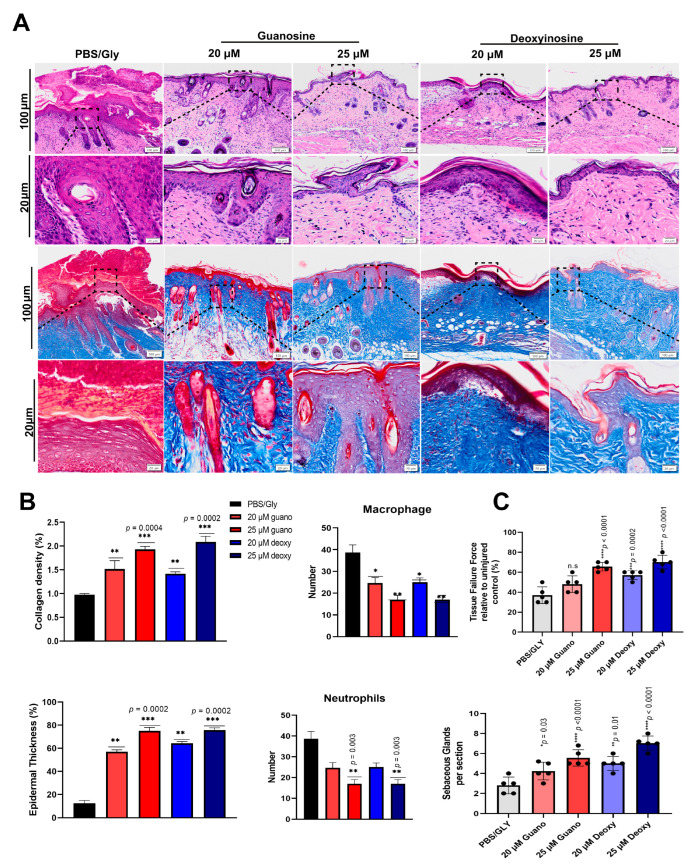
Representative images of H&E staining of inflammatory cells in wound tissues using guanosine and deoxyinosine. Guanosine and deoxyinosine affect the generation of extracellular matrix components (**A**). Histological analysis of fibroblasts and collagen fibers using Masson’s trichrome staining is shown. Tissues of wounds were obtained after 10 days post-healing. The scale bar represents 100 μm for the upper panel and 20 μm for the bottom panel. The relative quantification of epidermal thickness, collagen density macrophage infiltration, and the number of neutrophils is shown in the bottom panel (**B**). Data have been represented as mean ± SD, where *n* = 5/group and *** *p* < 0.001, ** *p* < 0.01, and * *p* < 0.05. Histological quantification of dermal thickness and wound healing parameters, including the number of sebaceous glands per section and fraction of ruptured skin evaluation while comparing with uninjured skin (**C**). Each point represents the average of two sections from two separate slides of one wound. Each dot point represents one animal and all the analysis was performed using a one-way analysis of variance (ANOVA). Data have been represented mean ± S.D, where *n* = 5. Data expressed as * *p* < 0.05, ** *p* < 0.001, *** *p* = 0.0001 and **** *p* < 0.0001.

The infiltration of neutrophils and macrophages was observed with tissue analysis through H&E staining and Masson’s trichrome staining (Figure 5A). The healing mechanism was evaluated through measuring the thickness of the epidermis and collagen density, as shown in Figure 5A, along with the quantification on the bottom panel. The infiltration of macrophages was significantly reduced in the groups consisting of 25 μM guanosine and 25 μM deoxyinosine compared with the control groups. Masson’s trichrome staining was used to stain the tissues for analyzing collagen fibers and fibroblasts in wound tissue. We found that a 25 μM dosage of both components (isolated from ceAF) increased the proportion of collagen fibers and fibroblasts compared with the control and the other two groups. Subsequently, inflammation-related factors, such as sebaceous glands, neutrophils, and number of macrophages, were analyzed using tissue samples (on the 10th day after euthanizing the animals). The expression profile of IL1-ß, released by activated macrophages, monocytes, and a set of dendritic cells post-inflammation, was also significantly increased in the 25 μM groups of both components (Figure 4).

## 3. Discussion

Only a few studies were conducted to explore the therapeutic potential of chick early amniotic fluid. We identified the potency of ceAF against skin injuries and purified active components that play roles in the healing of cutaneous wounds. We performed series of purification methods on ceAF to identify active components and eventually narrowed down the structural analogs of the purine-containing nucleosides guanosine and deoxyinosine. These molecules showed significant efficacies in embryogenesis, structural development, and metabolic reprogramming [16]. Purines have been known to provide a significant role in metabolic signaling, energy supplements, controlling the growth of cells, and contributing to the transport of sugars, along with donating phosphate groups in phosphorylation reactions. Studies have shown that purines could provide critical substrates for proliferative wound healing responses [17,18]. Given that in vitro and in vivo extracellular purine-containing nucleosides have a long-term and immediate trophic impact, including the stimulation of astrocytes and neural differentiation, morphogenesis, mitosis, apoptosis, and stimulation of trophic and growth factor synthesis [19], we were encouraged to test for the efficacies of guanosine and deoxyinosine in wound healing, and found that the optimum dosage for both components exist between 20–25 μM for keratinocytes (Figure 2A). However, it fluctuates for fibroblasts concerning guanosine.

We performed other in vitro assays, including wound healing (Figure 2) and trans-well migration assays (Figure 3A,B), to elucidate the influential role of these components. The in vitro findings were consistent with the murine in vivo models, where we topically applied these components mixed with glycerol (40%) and PBS (5% of 20× stock) on the cutaneous wounds (Figure 3C). The groups with 25 μM guanosine and deoxyinosine exhibited smooth epidermal skin in the given time frame compared with the control/vehicle group. The biochemical parameters, including collagen density, macrophage number, neutrophils, and epidermis thickness, were validated with H&E and Masson’s trichrome staining (Figure 5), which supported our findings. Furthermore, the mRNA and proteins were isolated from wound/healed samples to understand the underlying molecular mechanisms. Real-time PCR and western blotting revealed upregulated expression levels of proinflammatory molecules, growth factors, and cytokines (Figure 4). These results, and ceAF as a crude material, imply promising therapeutic applications of ceAF and its derived components.

Here, in our study, we first performed a series of purification processes to target the active compounds present inside ceAF. Since we already explored the healing abilities of ceAF when used as a whole compound [8] for wound healing as well as against tumorigenesis [9], these findings encouraged us to narrow down the active compounds. Once the active compounds were extracted, we utilized their promising healing properties on the wound healing phenomenon and designed this study. Our studies are unique in a way in that not much work has been performed on embryonic extracts isolated from avians as well as to study the impact of purines on the cutaneous wound healing phenomenon. Surveys in Denmark and UK reveal about 3–4 people with one or more wounds per 1000 population [20,21,22,23,24,25]. More studies are required to research various wounds, including accidental injuries, diabetic wounds, chronic ulcers, and amputations. Also, preclinical studies are required to access the wound healing parameters of different species, including animal and human skin samples. Exploring the underlying mechanisms involved in signaling pathways included in different phases of wound healing and more bioinformatic assessments are also required for more precise evaluation.

## 4. Materials and Methods

### 4.1. Ethics and Animals

All animal studies were performed under the guidelines of the National Institutes of Health’s Guide for the Care and Use of Laboratory Animals, and the experimental protocols were approved by the Research Ethics Committee of Zhejiang University (Permit #: ZJU20170013). C57BL/6 mice, specifically pathogen-free (SPF) (all 5-week-old males weighted around 20–26 g), were bought from the SIPPR-BK Lab Animals Co., Ltd. Shanghai, China [Certificate # SCK (hu) 2013-0016]. They were grouped randomly (5 mice/cage) and kept in the Animal Facility at Zhejiang University until the completion of the experiment. Institutional animal care uses committee-approved animal procedures.

### 4.2. Cell Lines

Cell lines, including HUFs (human uterine fibroblasts) and HaCaTs (human epidermal keratinocytes), were cultured in Dulbecco’s modified Eagle Medium (high glucose) (Gib-co™) supplemented with 10% fetal bovine serum (Gibco™). The medium was supplemented with antibiotics, including penicillin and streptomycin (Biological Industries) 100 U/mL. Cells were kept in the incubator (5% CO_2_ and 37 °C) for culturing and sub-culturing.

### 4.3. Chick Early Amniotic Fluid (ceAF) Preparation

Fertilized chick eggs were incubated at 38 ± 1 °C and 50% humidity, and ceAF was collected from eggs between 6–8 days. After centrifuging the samples at 2500× *g* for 20 min, supernatants were filtered over a 0.22 µm sterilization device (Millipore, Nantong, China) and stored in aliquots at −80 °C after quick freezing in liquid nitrogen. To make it easy for daubing on wounds made on lab mice, 0%, 5%, or 10% ceAF (*v*/*v*) was made to have a cream-like texture in 5% 20× PBS, 40% glycerol, and 50% distilled water. These creams were preserved at −30 °C for long-term storage.

### 4.4. Cell Culture and Differentiation

HaCaT and HUF cells, a gift from YU Faxing (Fudan University), was experimentally validated free of mycoplasma and assured to have a typical shape. Cells were cultured in DMEM (basal formula with 1% penicillin–streptomycin [GIBCO-Life Technologies, Bleiswijk, The Netherlands]) supplemented with 10% FBS (Capricorn, FSS500, Uruguay) or indicated doses of compounds. Cells were cultured at 37 °C in a humidified atmosphere of 5% CO_2_.

### 4.5. Effective Concentration (EC50) Assay

The effective concentration of compounds extracted from ceAF was generally assessed in a 96-well format using the cell counting Kit-8 (CCK-8) MedChemExpress^®®^ (Monmouth Junction, NJ, USA) assay. To determine the effective concentration of EC50 components (guanosine and deoxyinosine), 1000 cells were seeded in 96-well plates. After 24 h, different concentrations of 1, 5, 10, 15, and 25 μΜ of chemicals were administered to cells; the microplate reader counted the viability of cells at a 450 nm absorbance. The values were plotted on the dosage response curve using GraphPad prism software (version 9), and the EC50 value was calculated for both the HaCaT and HUF cell lines.

### 4.6. Cell Scratch Assay

HaCaT and HUF cells were seeded in a 6-well plate (Corning™, New York, NY, USA) and starved for 12 h. Later, guanosine in different fractions (HaCaT cells: 5, 10, 15, 20, and 25 μM and HUF cells: 2,4, 6, 8, and 10 μM, respectively) was supplemented on its EC50 values in the cells. Similarly, for deoxyinosine (HaCaT and HUF cells: 5, 10, 15, 20, and 25 μM), the components were used as a treatment. A confluent monolayer of the scratch was made, and micrographs were taken. For HUF cells, the time intervals were recorded as 0 and 24 h, respectively, whereas for HaCaT cells, an additional 48 h interval was recorded.

### 4.7. Trans-Well Migration Assay

To find out the migrations of cells after supplementing with different fractions of components (deoxyinosine and guanosine) and ceAF, we seeded HaCaT and HUF cells in 24-well plate-containing chambers (Corning™, USA). Cells were suspended at a density of 3 × 10^4^ cells in a 200 µL serum-free medium for the components guanosine and deoxyinosine. The indicated doses were as follows: for guanosine, HaCaT cells: 5, 10, 15, 20, and 25 μM, and HUF cells: 2,4, 6, 8, and 10 μM, and for deoxyinosine, HaCaT and HUF cells: 5, 10, 15, 20, and 25 μM. After 24 h, chambers were parted, and the cells present on the upper side of the membrane were wiped off with cotton buds. Cells that invaded the microporous membrane (8 µm diameter) were washed with PBS three times; fixation was performed with 4% paraformaldehyde for 30 min and stained with 0.1% crystal violet. The number of cells that trans-well-migrated was observed using a microscope (Olympus BX51, Tokyo, Japan).

### 4.8. RNA Extraction from Cells and cDNA Preparation

For isolating RNA from ceAF-treated cells, the cells were trypsinized and transferred in RNAse-free tubes following the protocol [23]. cDNA was prepared from isolating total RNA using a cDNA preparation kit (Takara Bio’s SYBR^®®^ Clontech™ Japan, Nagoya, Japan). RNase-free double distilled water, 4× *g* DNA wiper mix, and 1 µg total RNA were sequentially pipet-ted into a tube and incubated at 42 °C for 2 min. Then, 5X Hi Script Mix II was added, and the samples were kept at 50 °C for 15 min and 85 °C for 5 s before qPCR. cDNA was stored at −20 °C for future use. The sequence of primers used for the experiment has been mentioned in Table 2.

### 4.9. Animal Testing and Caring

C57BL/6 mice, specifically pathogen-free (SPF) (all 5-week-old males weighted around 20–26 g), were bought from the SIPPR-BK Lab Animals Co., Ltd. Shanghai, China [Certificate # SCK (hu) 2013-0016]. They were grouped randomly (5 mice/cage/group) and kept in the Animal Facility, Zhejiang University (Permit #: ZJU20170013) with a 12 h day/light cycle and water and rodent chow access. The number of animals used for this study were kept minimal in order to follow institutional ethical policies. The institutional animal care use committee approved the animal procedures.

### 4.10. Surgical Wound-Related Procedures

Strict guidelines and procedures were followed. Anesthesia (phentolamine; 8 mg/kg) was administered intraperitoneally. Procedures were conducted stepwise and aseptically: a full-thickness wound (10 × 10 mm) was excised from the dorsum of mice after hair removal; excessive panniculus carnosus layer’s shrinkage was controlled using silicon disks which were to the diameters of the wound’s size; interrupted sutures fixed the disks with no dressing applied. For guanosine and deoxyinosine, the components were supplemented with a cocktail (40% glycerol, 50% PBS, and different concentrations of chemicals) indicated as 20 and 25 μM. The control group contained only PBS and glycerol, named as PBS/Gly.

### 4.11. Dermal Histopathology

The mice were euthanized, and excised wound samples were collected and fixed in 10% formalin for at least five days and later processed. The specimens were embedded in paraffin, sectioned in 3 μm thickness, later mounted on glass slides, deparaffinized, and stained with hematoxylin–eosin and Masson’s trichrome. The density of inflammatory cells and blood vessels in the dermis was analyzed using the M-42 system [25] (Supplementary Figure S3). Random fields of the tissue were assessed and counted for particular sections with images taken using an optic microscope (Olympus, BX41).

### 4.12. Protein Extraction and Western Blotting

Aspirated media cells were rinsed with PBS twice and lyzed with HEPES lysis buffer (115 mM NaCl, 1.2 mM CaCl2, 1.2 mM MgCl2, 2.4 mM K2HPO4, 20 mM Hepes-KOH, pH 7.0, and 1% NP40) supplemented with protease and phosphatase inhibitors. Protein levels were normalized for SDS-PAGE loading. Protein expression was further normalized with an internal control (β-actin). Antibodies, including PDGF-A (#3174), TGF-ß (#3711), CCL5 (#2989), and VEGF (#2463) were purchased from Cell signaling technology™, Beverly, MA, USA. The lyzed protein load was first run on SDS -PAGE as 15 μL/well. Signals were detected using primary antibodies followed by HRP-conjugated secondary antibodies.

### 4.13. Quantification and Statistical Analysis

Numerical data were expressed as mean ± SEM and statistical analysis was performed using Prism GraphPad (version 9) (San Diego, CA, USA). One-way ANOVA, two-way ANOVA, Dunnett’s *T*-Test, Bonferroni post-hoc test, and the Student’s *t*-test were used to reveal significances. * *p* < 0.05, ** *p* < 0.01, and *** *p* < 0.001 were considered statistically significant. All in vitro experiments were independently repeated in triplicates.

## 5. Conclusions

ceAF plays a promising role in cutaneous wound healing. Our previous study already showed promising results of ceAF when applied topically [8]. This study is the extended work of the previous one where we extracted the components, guanosine and deoxyinosine, from ceAF and studied their potent role against cutaneous wounds. Our studies imply that the key components are the compounds guanosine and deoxyinosine, extracted after a series of modern cutting-edge purification techniques. However, more studies are required to study the promising role of these compounds, and which promising underlying signaling pathway plays a key role in regulating and secreting these compounds during embryogenesis and in the adult chick embryo.

## 6. Limitations

- Preclinical studies with volunteers and patients suffering from different types of wounds to explore if the same dose is consistently using the active components guanosine and deoxyinosine. These future studies are in the planning stage by the collaborative company.
- Conducting a study to compare the efficacies of guanosine and deoxyinosine separately and finding out one single target.
- Using advanced tools and technologies, such as RNA-seq and genome sequencing, to find what possible role these purines play in embryonic development.
- Finding out the possible adjuvants and compounds that can enhance the healing mechanism faster.

## Figures and Tables

**Table 1:** Effective concentrations of ceAF components using different cell lines.

Cell Line	Effective Concentration (EC_50_)	Component
HaCaT	14.08 μM	Guanosine
HUF	4.54 μM	
HaCaT	13.37μM	Deoxyinosine
HUF	15.7μM	

Abbreviations: HUF (human uterine fibroblast), and HaCaT (immortalized human keratinocyte).

**Table 2:** List of primers and their sequences.

Gene	Sequence F	Sequence R
*IL-1β*	GTATCACTCATTGTGGCTGTG	ATTTTGTCGTTGCTTGGTTCTC
*IL-10*	AGCTGAGAACCAAGACCCAGAC	AAGAAATCGATGACAGCGCC
*VEGF*	TGCTTCTGAGTTGCCCAGGA	TGGTTTCAATGGTGTGAGGACATAG
*TGF-β*	TTGCTTCAGCTCCACAGAGA	TGGTTGTAGAGGGCAAGGAC
*PDGF*	GCAACGAGGTGGTCAACTTC	GCAGGAATAGAACGGATGTGG
*GAPDH*	AGCCACATCGCTCAGACAC	GCCCAATACGACCAATCC

Abbreviations: *IL-1β* (interleukin 1 beta), *IL-10* (interleukin-10), *VEGF* (vascular endothelial growth factor), *TGF-β* (transforming growth factor-β), *PDGF* (platelet-derived growth factor), and *GAPDH* (glyceraldehyde-3-phosphate dehydrogenase).

## Data Availability

Data available in a publicly accessible repository.

## Supplementary Materials

The supporting information can be downloaded at: https://www.mdpi.com/article/10.3390/ijms241612817/s1.
