# Supplementary material for: Guanosine and Deoxyinosine Structural Analogs Extracted from Chick Early Amniotic Fluid Promote Cutaneous Wound Healing
Source: Int J Mol Sci. 2023 Aug 15;24(16):12817. doi: 10.3390/ijms241612817 (PMC10454561; doi:10.3390/ijms241612817)
Supplement: Supplementary file 1 [file ijms-24-12817-s001.zip › ijms-2514874-supplementary.pdf]

Supplementary Information

A

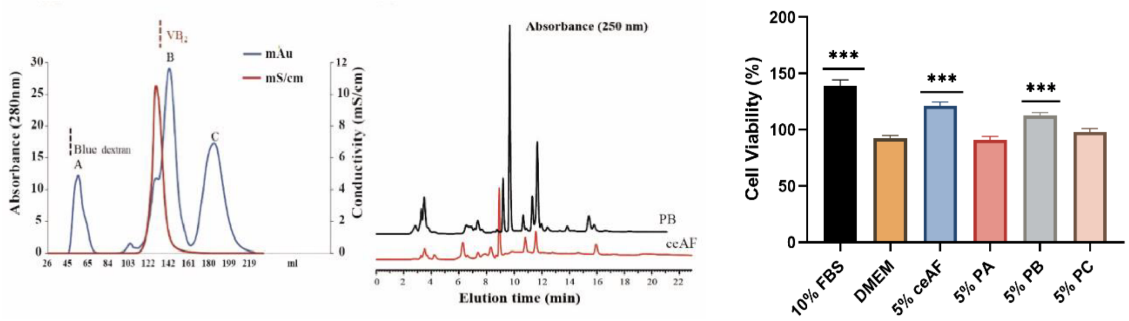

B

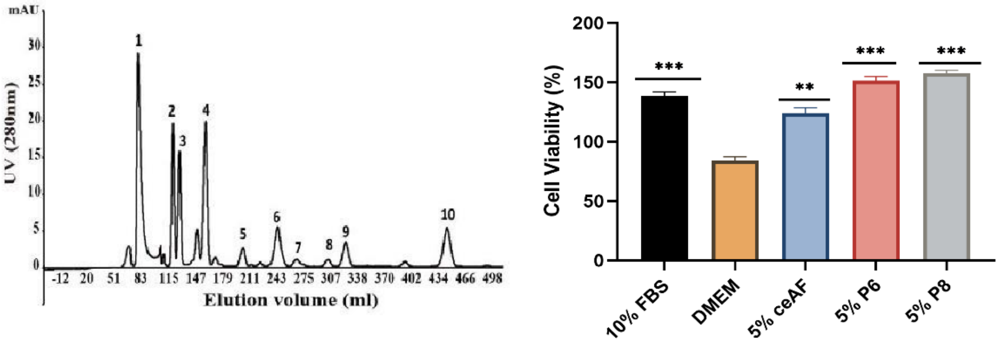

C

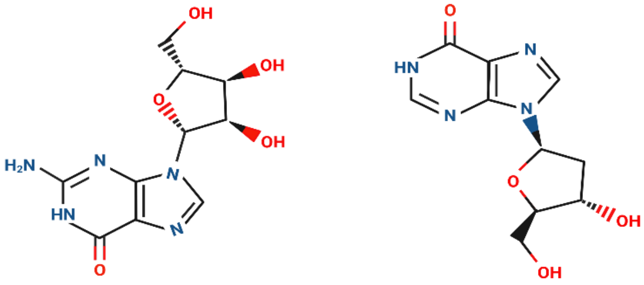

Figure S1. Purification of active components of ceAF using liquid chromatography and analyzing the peaks. Three peaks (PA, PB and PC) were separated on the sizing column (A); and the size of PB is close to that of VB12 which is close to 1.4 KD, the elution profile of which is in red. Comparative analysis of ceAF and peak B (PB) using ion exchange column is shown in. Cell viability assay using PA/PB/PC with native ceAF as an active control is shown. Only PB shows similar activity as compared with that of ceAF. Data represents as mean ±SD whereas $^{**}P<0.01$ and $^{***}P<0.001$. B) Separation of active components from PB obtained after initial purification of ceAF. C) Ten separate peaks were obtained. Cell viability assay was performed on P8 and P6 on HaCaT cells comparing with the native ceAF indicating the efficient biological activity of these peaks

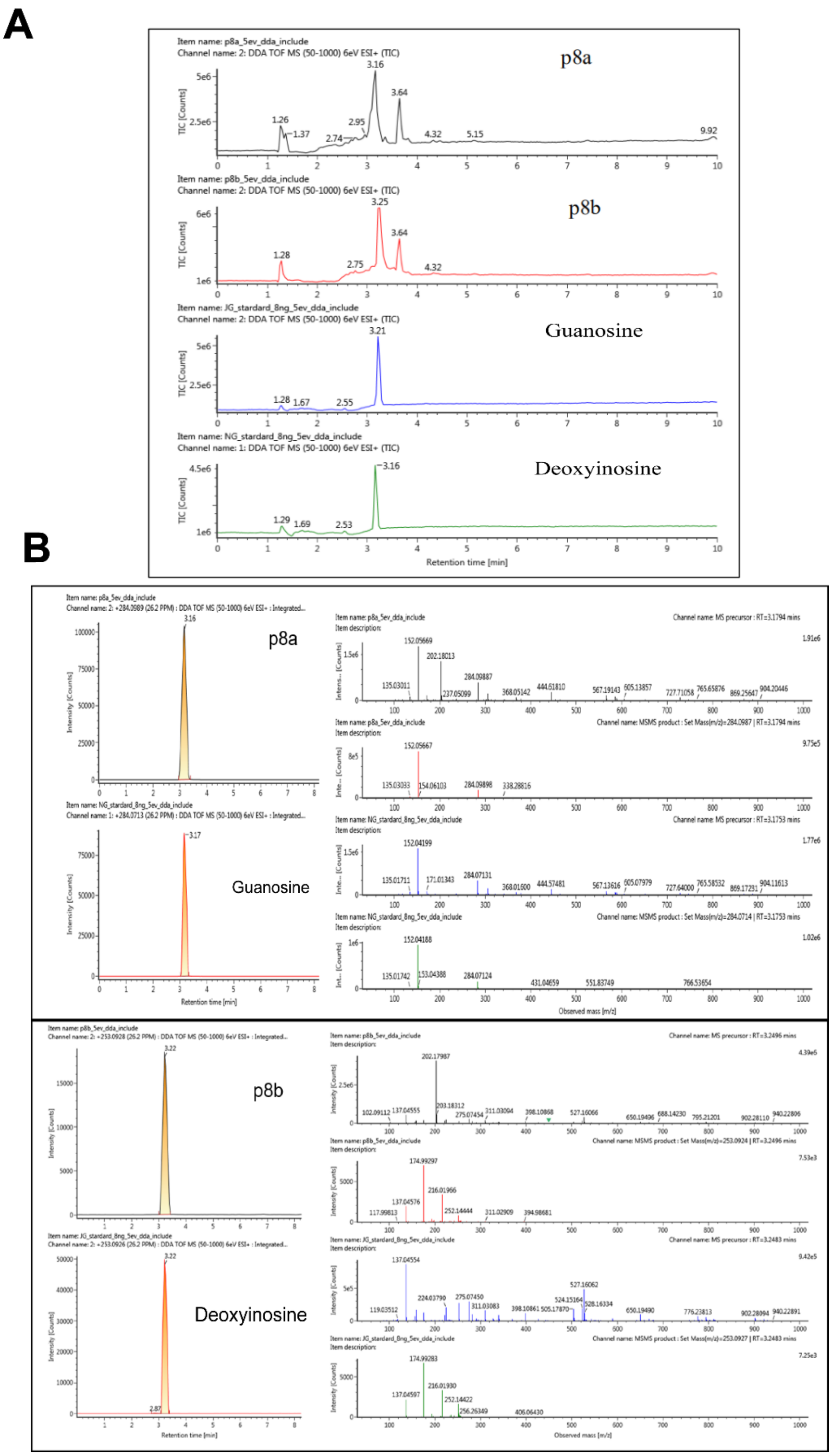

Figure S2. (A,B) TIC (total ion chromatogram) peaks of p8a, p8b, guanosine (reference) and deoxyinosine (reference)

Inflammatory Response and Healing Parameters for Cutaneous Wound Healing

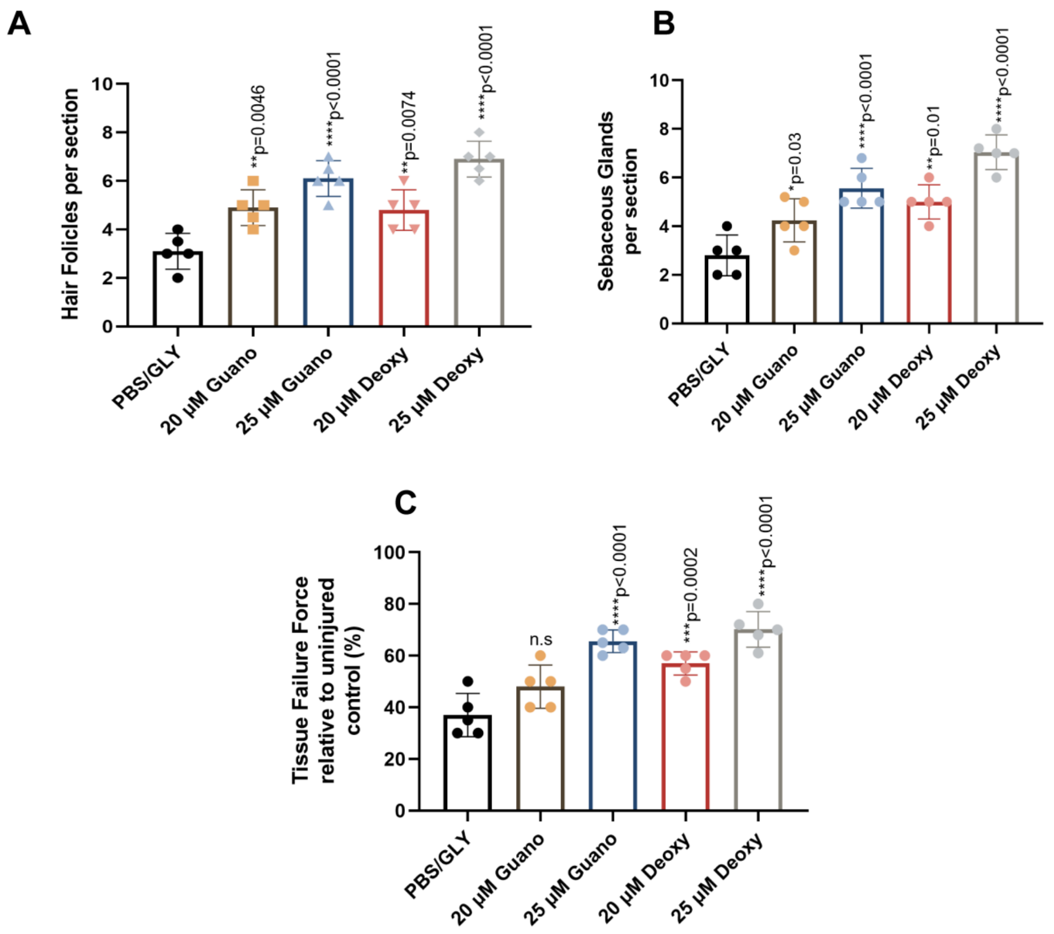

Figure S3. Assessment of inflammatory response and healing parameters in wound healing. (A-C) Histological quantification of dermal thickness and wound healing parameters including (A) Number of hair follicles per section, (B) Number of sebaceous glands per section and fraction of ruptured skin evaluation while comparing with uninjured skin (C). Each point represents the average of two sections from two separate slides of one wound. Each data point represents one animal and all the analysis is by one-way analysis of variance (ANOVA). Data represents as mean ± S.D. where N=5. Data expressed as *p<0.05, **p<0.001, ***p=0.0001 and ****p<0.0001
